# Masticatory Ability for a Single Implant Mandibular Overdenture Retained by Two Different Attachments: A Randomized Controlled Trial

**DOI:** 10.1155/2021/1632848

**Published:** 2021-09-08

**Authors:** Marwa Abdel AaL, Amr Naguib, Ahmed Salah, Karim Foda, Nora Sheta, Nouran Abdel Nabi

**Affiliations:** Department of Prosthodontics, Faculty of Dentistry, Cairo University, Giza, Egypt

## Abstract

**Objective:**

The aim of this randomized clinical trial was to compare the masticatory ability subjectively between ball and Cendres+Métaux Locator (CM-LOC) attachment for a single implant retained mandibular overdenture throughout a 24-month follow-up period.

**Materials and Methods:**

Eighty completely edentulous patients were recruited. All patients received new complete dentures, and masticatory ability was recorded using a questionnaire (baseline record). All patients received a single implant in the midline of the completely edentulous mandible. After 3-month healing period, patients were randomized using sealed envelopes into two groups: ball or CM-LOC attachment. The same masticatory ability questionnaire was used to record masticatory ability for both groups after 2 weeks of pickup and 3-, 6-, 9-, 12-, and 24-month follow-up. Comparison between the study groups was done using Mann–Whitney *U* test for independent samples. Two-sided *P* values less than 0.05 were considered statistically significant.

**Results:**

The mean masticatory scores improved for both attachments, with no statistically significant difference between them throughout the 24-month follow-up. The CM-LOC attachment group showed a greater improvement change in masticatory ability after 6- and 12-month follow-up (−12.47 ± 12.006, −11.46 ± 14.625; *P*=0.826), while the ball attachment group showed a slight improvement after the 24-month follow-up (−11.72 ± 12.368, −10.88 ± 11.963; *P*=0.778).

**Conclusion:**

Single implant retained mandibular overdenture improved masticatory ability subjectively with no significant difference between both attachments used although the ball attachment showed better masticatory ability scores after 24-month follow-up.

## 1. Introduction

The McGill consensus 2002 and York consensus 2009 concluded that two implants installed in the mandible were considered the standard of care for completely edentulous patients [1–3]. To reduce the cost and time of treatment, Cordioli et al., introduced the concept of a single implant retained mandibular overdenture which provided an alternative treatment option for the elderly population [4]. The single implant retained mandibular overdenture is considered a cost-effective treatment option which has demonstrated medium- to long-term survival rates [4–6]. Harder et al. 2011 and Cheng et al. 2012 reported that a single implant installed in the midline is an efficient treatment option like two implants installed in the mandible [7, 8]. Furthermore, Padmanabhan et al. 2020 conducted a systematic review and concluded that a single implant retained mandibular overdenture is a cost-effective minimally invasive treatment that can restore function and esthetics for completely edentulous patients with minimal prosthetic complications and high survival rates [9].

The choice of the attachment system for the implant retained overdentures is considered of great importance as it has an impact on the overall patient satisfaction and clinical success [10]. Ball and socket attachment is the most popular unsplinted attachment used to retain a mandibular overdenture, because of its simplicity and cost effectiveness, and it has an impact on patient satisfaction [11]. Previous studies reported that a single implant retained overdenture using a ball or locator attachment to support an overdenture showed satisfactory outcomes [4, 12–18].

The Cendres+Métaux Locator attachment (CM-LOC) is a newly introduced attachment made from polyetherketoneketone (PEKK), which is a member of the polyaryletherketones (PAEKs). Polyaryletherketones have the advantages of high chemical and mechanical resistance to wear and high tensile, fatigue, and flexural strengths [19]. Passia et al., Choi et al., and Maniewicz et al. in 2020 concluded that the combination of a titanium matrix and a matrix made from polyetherketoneketone seems to be a promising combination for long-term retention, with parallel and tilted implants [20–22].

One of the important goals of prosthetic dentistry is to restore the masticatory function of the oral cavity. Masticatory function is defined as the ability to masticate food. It can be divided into two subdomains: The first is the objective and quantifiable capacity to mix and chew solid food and is generally called “masticatory performance.” Masticatory performance is measured through comminution tests, in which brittle food like nuts are masticated for a number of cycles into small particles and then the masticatory performance is quantified by assessing the size of the small particles [23]. The second subdomain is termed “masticatory ability” and it subjectively assesses masticatory function in the patients' opinion through quantified questionnaires, which are specifically designed to assess masticatory ability, while more generalized questionnaires are used [24]. Masticatory function considers both the quantitative (objective) and qualitative (subjective) assessment which correlate weakly or not at all [25–27]. Several authors reported that objective measurements are important during planning the treatment and determining the effects of prosthetic treatment [25–27]. Masticatory function is a multifactorial phenomenon, so besides the objective tests used, the individual experience of the patients should be greatly considered to determine the masticatory function of any given prosthetic treatment.

The aim of this randomized clinical trial was to compare the masticatory ability subjectively between ball and CM-LOC attachment for a single implant retained mandibular overdenture throughout a 24-month follow-up period.

## 2. Materials and Methods

The study proposal was approved by the Ethical Committee of Prosthodontics Department, Faculty of Dentistry, Cairo University, on June 13, 2016 (Ethical Approval Number: 16/6/10), and was registered at https://www.pactr.org/, Trial Number: PACTR201803003085193.

### 2.1. Trial Design

This is a two-arm randomized clinical trial, with an allocation ratio of 1 : 1.

#### 2.1.1. Participants (Eligibility Criteria)

Eighty fully completely edentulous patients, seeking to improve the retention of their mandibular dentures and willing to install a single midline implant for that purpose, were recruited.

All included patients were recruited following strict inclusion criteria: age of 50–69; glycosylated hemoglobin level <8; and only classes II and III according to Thomas McGarry 1999 [28]. Minimum of 5 mm bone width had to be present in the anterior area of the mandible directly or after minimum plateauing. Patients with any condition that may contraindicate implant placement were excluded. An informed consent had to be signed by all patients before implant installation.

### 2.2. Sample Size Calculation

In the present clinical trial, the longitudinal observational study which was carried out by Schuster et al. [29] was used to calculate the sample size. The mean and standard deviation for the functional limitation domain of the OHIP-EDENT of the mandibular implant retained overdenture using stud attachment which was reported in the study by Schuster et al. [29] were 1.52 ± 1.55, the minimum clinically important difference based on expert opinion for the CM-LOC group was calculated and was equal to = 1, the alpha significance was 0.05, and the power of the study was 80%. Sample size calculation was carried out using the PS software, using independent *t*-test. The sample size calculated was 39 patients in each group, hence a total of 78 patients for both groups.

### 2.3. Intervention

**Figure 1:**
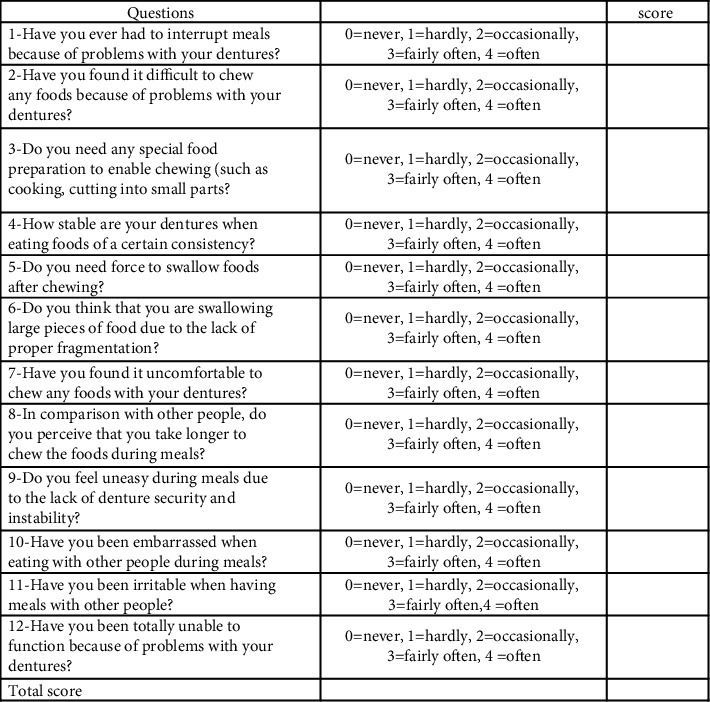
Masticatory ability [30] consists of 12 questions, and each question has options with the best score being 0 and worst score being 4; a total score was calculated for each patient.

All patients had newly constructed maxillary and mandibular complete dentures. Patients were allowed to adapt to their newly constructed dentures for a 6 week period. A masticatory ability questionnaire was used to record the masticatory ability for all patients after denture adaptation (Figure 1). The masticatory ability (R) chart used in this study consisted of 12 questions, each with scores from 0 to 4, where 0 means “never” (no problem) and 4 means “always” (with problems). A score for each question was recorded, and then a total score for the 12 questions was added. The lower the score, the greater the masticatory ability [30] (Figure 1). This masticatory ability chart used in this clinical trial was previously used in a prospective study by Pocztaruk et al. [30] to evaluate the satisfaction level and masticatory capacity of edentulous patients by applying questions from indexes OHIP [31] and OHIP-EDENT [32] during the different phases of rehabilitation with dental implants. Karim Foda (KF) and Ahmed Salah (AS) were responsible for asking the patients to fill the charts, and each of them were responsible for following the same group of patients throughout the 24-month follow-up period.

### 2.4. Implant Installation

A small crestal incision was made in the central incisor area. All implants installed in this study were of 3.7 mm diameter and 10 mm length (Zimmer dental implants, ZDI, Tapered Screw-Vent, Indiana, USA). One prosthodontist, Marwa Abdel Aal, installed all implants in this study. Drilling was carried out according to the manufacturer's instructions, using the Zimmer kit. All installed implants were left to heal for a 3 month period, and the patient's denture was properly relieved using a soft liner (Soft-Liner, GC Corporation, Tokyo, Japan).

At the end of the healing phase, 6 patients reported implant failure, and 3 patients were considered as dropouts. A total of 71 patients were then ready to receive the attachment.

### 2.5. Description of the Study Sample

**Figure 2:**
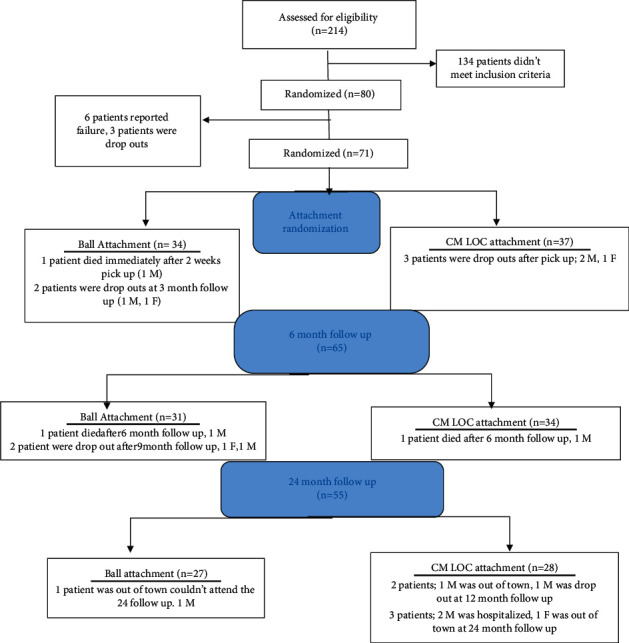
Consort flow diagram showing dropouts and including patients throughout the 24-month follow-up period (M: male; F: female).

Two hundred and fourteen patients were recruited; 134 patients were excluded as they did not meet the inclusion criteria. Eighty patients were then included in the present trial: 56 males and 23 females, with a mean age of 62.5 for males and 59.6 for females. Thirty-four (34) patients were assigned to the ball attachment group, 21 males and 13 females, and 37 patients to the CM-LOC attachment group, 29 males and 8 females (Figure 2 and Table 1).

### 2.6. Randomization and Allocation Concealment

**Figure 3:**
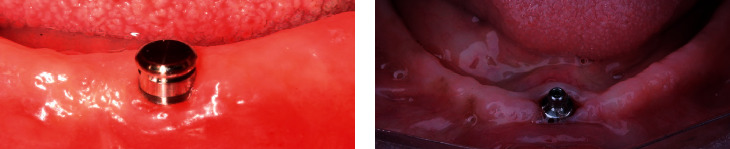
(a) CM-LOC attachment. (b) Ball attachment.

Two attachments were used in this study: ball attachment with a nylon matrix (Zimmer dental implants) and CM-LOC attachment with a PEKK matrix (Cendres+Métaux). The Cendres+Métaux Locator (CM-LOC) attachment system comprises a male implant straight abutment with a gingival cuff height ranging from 1 to 5 mm (Figure 3(a)) and a denture attachment housing containing a retentive insert. CM-LOC abutments are made of titanium grade V and have a noncoated surface. They are both directly tightened onto the implant using a specific screwdriver. The housings are made either of polyetherketoneketone (PEKK, Pekkton, Cendres+Métaux SA) or of titanium, with a lodged Pekkton retentive insert available in different strengths; in this study the “medium” (green) retentive insert was used.

The ball attachment (Zimmer dental implants) compromises a male abutment with a gingival cuff height of 2 mm and 4 mm. The abutment was screwed onto the underlying implant using a specific screwdriver. The housings are made of titanium with a nylon transparent retentive insert that was supplied from Zimmer Company with the ball abutment (Figure 3(b)).

The height of each attachment used in the following phases of the study was not standardized as it depends upon the amount of mucosa present after healing, which was different for each patient.

After the 3-month healing period, patients were randomized using sealed envelopes to receive either ball or CM-LOC attachment. Randomization and allocation concealment were carried out by Amr Naguib (AN), as he was responsible for preparing the envelopes used in randomization. Seventy-one patients were randomized into the two groups as the sealed envelopes were prepared at the beginning of the study before dropout: 34 patients in the ball group and 37 patients in the CM-LOC group (Figure 2 and Table 1).

### 2.7. Attachment Installation and Pickup

Both attachments were screwed to the implant with a torque of 30 N/cm, each with the corresponding matrix on top of it. The mandibular denture was then modified to receive the housing by drilling a small hole in the area corresponding to the attachment to allow for the escape of the excess acrylic resin, and a red die was placed on the fitting surface of the modified denture to ensure that there was no interference between the acrylic resin and the attachment matrix. The mandibular denture was checked for proper seating, and the occlusion of the maxillary denture was properly checked.

All undercuts present in both attachments were blocked before pickup procedure. The denture was then properly seated in place, and then the fitting surface of the denture was finished and polished. The patient was then asked to bite gently in the centric occlusion.

**Figure 4:**
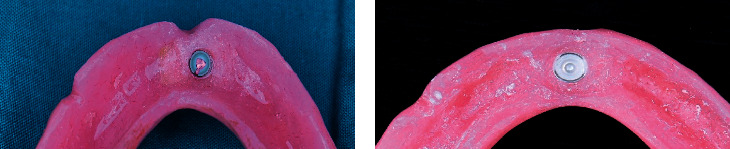
(a) PEKK matrix after pickup. (b) Nylon matrix after pickup.

After complete setting of LuxaPick-up material, the denture was removed and pickup of the matrix was checked (Figures 4(a) and 4(b)). All excess was removed and then polished. Patients were recalled 3 days after pickup to check if there are any premature contacts or areas that required relief. This procedure was carried out for both attachments used in this study.

The same masticatory ability questionnaire was used to record patient satisfaction for both groups of patients at the following intervals: 2 weeks after pickup and after 3-, 6-, 9-, 12-, and 24-month follow-up (Figure 1).

The number of patients who attended the 2 week follow-up was 33 for the ball attachment and 34 for the CM-LOC attachment group (Tables 2 and 3). At 12-month follow-up, the total number of patients was 59: 28 for the ball attachment and 31 for the CM-LOC attachment (Tables 2 and 3). At 24-month follow-up, the total number of patients was 55: 27 for the ball attachment group and 28 for the CM-LOC group (Figure 2 and Tables 2 and 3).

Data were statistically described in terms of mean ± standard deviation (±SD). Numerical data were tested for the normal assumption using Kolmogorov–Smirnov test. Comparison between the study groups was done using Mann–Whitney *U* test for independent samples. Two-sided *P* values less than 0.05 were considered statistically significant. All statistical calculations were done using the computer program IBM SPSS (Statistical Package for the Social Sciences; IBM Corp., Armonk, NY, USA) release 22 for Microsoft Windows.

## 3. Results

**Figure 5:**
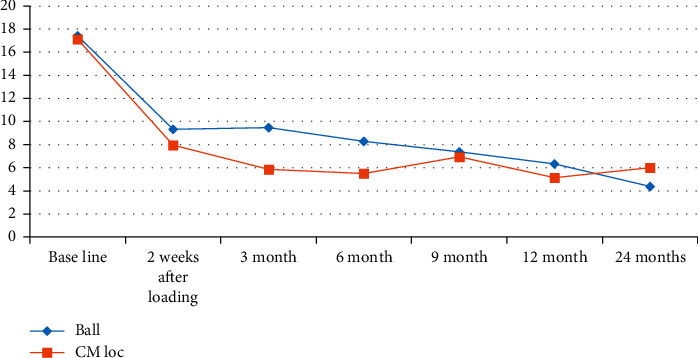
Mean scores for masticatory ability for the two attachments used, ball and CM-LOC attachment, throughout the 24-month follow-up interval.

There was no statistically significant difference for the mean masticatory ability scores between the two groups, ball and CM-LOC attachment, throughout the 24-month follow-up. At baseline (complete denture), the masticatory ability score was nearly equal for both groups and was the highest score recorded throughout the different follow-up intervals (17.43 ± 11.488, 17.15 ± 10.581; *P*=0.962), which denotes poor masticatory ability (Table 4 and Figure 5). Following the installation of a single implant in the midline of the edentulous mandible, the masticatory ability scores improved for both groups of patients throughout the 24-month follow-up period. The CM-LOC attachment showed a slightly better masticatory ability after 2 week follow-up till 12-month follow-up. At 9-month follow-up, the mean masticatory ability scores for both attachments were nearly equal (7.38 ± 9.034, 6.96 ± 10.017; *P*=0.813) (Table 4 and Figure 5). However, at 24-month follow-up, patients with ball attachment showed an improvement in masticatory ability when compared to the CM-LOC group (4.38 ± 5.558, 6 ± 9.087; *P*=0.884) (Table 4 and Figure 5).

When having a closer look at the changes in the mean masticatory ability scores for patients with their complete dentures and then at 6-, 12-, and 24-month follow-up after installation of a single implant in the midline of a completely edentulous mandible, we found that there was no statistically significant difference between the two groups of patients, but there was an improvement in mean masticatory ability scores for patients with ball and CM-LOC attachment after implant installation, as negative values denote more improvement (Table 5. Patients with CM-LOC attachment showed a greater improvement in masticatory ability scores at the first 6- and 12-month follow-up (−12.47 ± 12.006, −11.46 ± 14.625; *P*=0.826), while patients with ball attachment have shown better improvement in mean masticatory ability scores after 24-month follow-up (−11.72 ± 12.368, −10.88 ± 11.963; *P*=0.778) (Table 5).

## 4. Discussion

Edentulism is considered one of the most common diseases that affect the oral health of the elderly population. Alveolar ridge resorption that follows tooth extraction will consequently result in reducing the area supporting the prosthesis which is more encountered in the mandible [33]. This consequence of edentulism will greatly affect the retention and stability of complete dentures constructed for edentulous patients [34]. Studies concluded that the decrease in retention and stability of the mandibular denture was considered one of the major causes of dissatisfaction and will have an impact on the oral health related quality of life (OHRQoL) [35]. Locker et al. defined OHRQoL as the extent to which oral disorders have an impact on the functioning and psychosocial well-being of patients [36]. Recently, OHRQoL is considered a crucial factor for the assessment of the success of implant retained mandibular overdentures [7, 37, 38]. That was the reason that, in the present clinical randomized trial, questions were adapted from the OHRQoL and the validated indexes OHIP [31] and OHIP-EDENT [32] were used to evaluate the masticatory ability of patients with a single implant retained mandibular overdenture.

Masticatory ability describes the patient's own opinion of their ability to eat food, which is considered a subjective assessment of the masticatory function. Results of the following trials revealed that the masticatory ability improved after the installation of a single implant in the midline of the mandibular completely edentulous ridge, mainly due to the increase in retention and stability offered by the single implant retained overdenture when compared to the patient's previous complete denture. This result also comes in agreement with several studies that concluded that implant retained mandibular overdenture improved masticatory ability and patient satisfaction [39–44]. In addition, several studies reported that single implant retained mandibular overdenture improved the masticatory function of elderly patients [42,44–48].

Patients with ACP Class I were not included in this study because, according to McGarry [28], the posterior bone height of the mandible range could be greater than 21 mm, so those patients will most probably be satisfied with their conventional mandibular complete dentures and will not need implant installation to improve their retention. Patients with ACP Class IV were also excluded because their posterior bone will offer little horizontal stability and so will require installation of two or more implants to improve retention of their mandibular denture. Therefore, only patients with ACP Class II and III were included.

The masticatory ability scores improved after implant installation for patients in both groups throughout the 24-month follow-up period with no significant difference between them, although the CM-LOC group of patients showed a slight improvement in masticatory ability when compared to the ball attachment group in the first 12-month follow-up. There seems to be some difference in the mode of action between the two attachments due to the difference in the retentive inserts, as the ball attachment has a retentive insert made of nylon, and the CM-LOC has a retentive insert made of PEKK. Several in vitro studies have reported the consistent retentive properties of the CM-LOC attachment [16–19], although very few clinical studies reported the performance of this attachment. It was noticed that patients in the CM-LOC group have required the change of the PEKK retentive matrix after a 9-month follow-up period; that was the reason why the masticatory ability was nearly equal in both groups at that follow-up period as patients with CM-LOC attachment had a new PEKK retentive cap which was actually comparable to those of the ball attachment. While patients with ball attachment requested the change in retentive nylon cap after a 12- to 14-month follow-up, it seems that the CM-LOC attachment might lose retention faster than the ball attachment which could have an impact on the masticatory ability. That could also explain why, at the 24-month follow-up period, the masticatory ability was slightly greater for patients with ball attachment than the CM-LOC attachment group. Another important factor that has to be considered is the incidence of fracture of the lower implant retained overdenture, which is a widespread incidence due to insufficient space to accommodate the height of the attachment [49]. Most fractures occurred in the CM-LOC group of patients after 12-month follow-up and that was mainly because the CM-LOC attachment had a greater height than the ball attachment, so probably that had negatively affected he masticatory ability in the 24-month follow-up period for the CM-LOC attachment.

## 5. Conclusion

Single implant retained mandibular overdenture improved masticatory ability subjectively when compared to complete denture irrespective of the type of attachment used. Both the ball and CM-LOC attachments improved the masticatory ability of a single implant retained denture with no statistically significant differences between them, despite the fact that the ball attachment showed a slight improvement after 24-month follow-up.

### 5.1. Limitations and Recommendations

One of the limitations of this study is that although the subjective evaluation of masticatory ability is important to help evaluate the masticatory function, some patients will acquire some form of adaptation and accommodation to the degradation in masticatory ability and will develop a positive view of their masticatory ability; they will perceive their masticatory ability to be good, even though their ability to process some food types is inadequate, which results in even the avoidance of some types of food. Therefore, it is recommended that both subjective and objective masticatory ability should be evaluated together with the prosthetic maintenance, as it seems that there is a relation between the incidences of changes of retentive insert and fracture of lower dentures which will consequently affect masticatory ability.

## Figures and Tables

**Table 1:** Demographic information of all participants.

	All participants	Participants (after 3-month healing period)	Ball attachment	CM-LOC attachment
Number	80	71	34	37

*Age (years)*				
Mean Minimum Maximum	62.5 50 69	60.5 50 69	58.3 50 69	61 50 69

*Sex (n) (%)*				
Male Female	57 (71.25%) 23 (28.6%)	50 (70.4%) 21 (29.6%)	21 (61.7%) 13 (38.2%)	29 (78.3%) 8 (21.6%)

*Class II (according to McGarry) (n)*				
Male (*n*) Female (*n*)	27 3	24 3	9 2	15 1

*Class III (according to McGarry) (n)*				
Male (*n*) female (*n*)	30 20	26 18	12 11	14 7

**Table 2:** Number of patients after randomization into the ball attachment group.

	Dropouts	Number of patients		Total number of patients
		M	F	
Number (beginning of randomization)		21	13	34
Patients who did not attend 2 weeks after pickup	1 patient died (M)	20	13	33
Patients who did not attend 3-month follow-up	2 patients dropped out (1 M; 1 F)	19	12	31
Patients who did not attend 6-month follow-up	1 patient died (M)	18	12	30
Patients who did not attend 9-month follow-up	2 patients dropped out (1 M; 1 F)	17	11	28
Patients who did not attend 12-month follow-up	No dropouts	17	11	28
Patients who did not attend 24-month follow-up	1 patient was out of town (M)	16	11	27

M: male, F: female.

**Table 3:** Number of patients after randomization for the CM-LOC attachment group.

	Dropouts	Number of patients		Total number of patients
		M	F	
Number (beginning of randomization)		29	8	37
Patients who did not 2 weeks after pickup	3 patients dropped out (2 M; 1 F)	27	7	34
Patients who did not attend 3-month follow-up	No dropouts	27	7	34
Patients who did not attend 6-month follow-up	1 patient died (M)	26	7	33
Patients who did not attend 9-month follow-up	No dropouts	26	7	33
Patients who did not attend 12-month follow-up	1 patient was out of town (M); 1 patient dropped out (M)	24	7	31
Patients who did not attend 24-month follow-up	2 patients were hospitalized (M); 1 patient was out of town (F)	22	6	28

M: male, F: female

**Table 4:** Mean scores and standard deviation of masticatory ability at baseline; 2 weeks from loading; and 3-, 6-, 9-, 12-, and 24-month follow-up, for patients of the ball and CM-LOC attachment group.

Group		Baseline (complete enture)	2 weeks from pickup	3-month follow-up	6-month follow-up	9-month follow-up	12-month follow-up	24-month follow-up
Ball	Mean	17.43	9.33	9.46	8.30	7.38	6.32	4.38
	SD	11.488	8.360	10.9	11.7	9.034	8.743	5.558
CM-LOC	Mean	17.15	7.95	5.88	5.52	6.96	5.13	6
	SD	10.581	8.936	6.058	6.674	10.017	7.566	9.078
	*P* value	0.962	0.764	0.631	0.835	0.813	0.628	0.884

SD: standard deviation. *P* ≤ 0.05 is considered statistically significant.

**Table 5:** Mean scores and standard deviation of change in masticatory ability from baseline to 6-, 12-, and 24-month follow-up for patients of the ball and CM-LOC attachment group.

Group		Baseline–6-month follow-up	Baseline–12-month follow-up	Baseline–24-month follow-up
Ball	Mean	−9.42	−11.46	−11.72
	SD	12.426	14.625	12.368
CM-LOC	Mean	−10.86	−12.47	−10.88
	SD	9.775	12.006	11.963
	*P* value	0.774	0.826	0.778

SD: standard deviation. *P* ≤ 0.05 is considered statistically significant.

## Data Availability

A protocol for the randomized clinical study is available from the corresponding author and will be sent when required.
